# Glances at the Hospitals

**Published:** 1903-05-23

**Authors:** 


### GLANCES AT THE HOSPITALS.

### ROYAL INFIRMARY, DUMFRIES.

The ordinary income was £3,975, and the ordinary expenditure was £4,163, showing a deficiency of income of about £187 on the year's working; but this is more than accounted for by the facts that provisions cost £174, and wages £99 in excess of last year. Turning to the extraordinary income, we find a better state of things. The sum of £1,065 was received, and only £694 expended. Various improvements have been carried out in the kitchen, operating-room, mortuary, etc. The number of patients treated in the infirmary was 758. The average duration of residence was 36 days, the cost per patient was £5 9s. 10d., the cost per bed occupied was £57, and the average cost per patient per day was 3s. The medical and surgical tables are clearly arranged. There were 16 cases of pneumonia, of whom 4 died; 53 cases of phthisis, of whom 13 were cured, 16 relieved, 6 not improved, 4 died, and 10 remained under treatment. Of 4 cases of fracture of base of the skull, 2 were cured, and 2 remained under treatment. Of 4 amputations of the thigh, 3 recovered and 1 died. The operation for the radical cure of hernia was performed 12 times, all being successful.

### YORK COUNTY HOSPITAL.

From the 162nd annual report we learn that the ordinary income for last year was £6,568, and legacies available as income £368, making a total of £6,937, and the expenditure was £6,950. There has been a decrease of £734 in the Ladies' County Hospital Fund, and of £300 in the donations, which, we hope, will be made up next year to their former amount. There were 99 patients in residence at the beginning of the year, and 1,221 were admitted during the year showing a total of 1,320 under treatment. The average duration of each patient's stay was 28 days, the daily average number resident was 97, the mortality was 6·1 per cent., but 24 died within 48 hours of admission. Of 34 cases of acute rheumatism one died, of 20 cases of pneumonia 3 died, of 17 cases of enteric fever 6 died. There were 3 amputations of the thigh and all recovered. Five cases of strangulated hernia were operated on and all recovered, and so with 4 operations for the radical cure of hernia. Altogether there were 563 operations. The anæsthetic table shows that chloroform was administered 357 times, ACE mixture 41, chloroform and ACE mixture 42, ether 17, nitrous oxide 12, nitrous oxide and ether 21, cocaine and ethyl chloride 95.

### MOUNT VERNON HOSPITAL FOR CONSUMPTION AT HAMPSTEAD AND NORTHWOOD.

The total expenditure was £10,902, and at the end of the year there was a deficit of £2,469. There was a falling off in subscriptions to the amount of £220, and this is to be deplored for, as the committee truly say, these annual subscriptions are the only trustworthy income of the charity They appeal for at least £5,000 a year under this heading The donations have also fallen from £2,898 to £2,365. The out-patients' department at 7 Fitzroy Square has been enlarged, and further improvements are required for which a sum of £4,500 will be necessary. During the year 547 patients were admitted as in-patients, the daily average number of beds occupied was 92, and the average residence of each patient was 61 days. The number of out-patients was 4,962. Applications for admission are received from almost every county in England. The hospital has lately increased the number of beds, and it is to be hoped that the appeal for funds will meet with a generous response. There are no medical statistics in the report, which we look upon as a serious omission.

### STROUD GENERAL HOSPITAL.

The receipts amounted to £1,969 and the expenditure to £1,984. It is worthy of notice that the workpeople's subscriptions have increased from £432 to £494. We are glad to mention this fact, as it shows what can be done in a small district like Stroud, and it should prove an incentive at other hospitals where this source of income is too often neglected. There were 12 patients in the hospital on January 1st., and 331 were admitted during the year, making a total of 343 under treatment. Of these 238 were discharged cured, 56 discharged relieved, 9 not relieved, 20 died, and 20 remained in the hospital. The average residence in the hospital was nearly 22 days. The medical and surgical statistics are condensed on one page, and merely show the numbers suffering from particular diseases, but give no details as to the result of treatment. Anæsthetics were administered 216 times, but their nature is not specified. We hope that the next report will convey more information on these points as comparison with kindred institutions cannot be satisfactorily made unless detailed information is given.

### NORTHAMPTON GENERAL INFIRMARY.

The ordinary income was £7,860, and the ordinary expenditure was £8,125. Many improvements and additions have been carried out during the year. For instance, a pathological laboratory and an ophthalmic room have been fitted up, and the old isolation hospital has been temporarily adapted for the open-air treatment of consumption. The infirmary having been erected before the principles of hospital architecture were understood was daily becoming more antiquated and unsuited for its purpose. The governors therefore wisely decided to build two new blocks and to convert the old building into a nurses' home, medical library, pathological museum, etc. The cost of the entire work will be about £30,000, of which £14,800 had been subscribed on the date of the report. The committee state that the new pavilions will be "of the most modern and complete description." We believe that the necessity for this new work was imperative if the infirmary was to hold its own among similar institutions, and we cannot doubt that the inhabitants of the town and county will respond cheerfully and largely so that the new building may be opened free of debt. The committee appeal for funds, and they say that when "the surrounding districts realise that they have a county hospital that they may justly be proud of, their appeal will not be in vain." We earnestly hope so. On January 1st the hospital contained 130 patients, 1,516 were admitted during the year making a total of 1,646. This number does not include 408 supplied with instruments, and the out-patients amounted to 10,522. Of the in-patients 901 were cured, 169 relieved, 14 were made out-patients, 60 discharged not relieved, 27 for various reasons, 73 died, and 130 remained in the house. The ordinary medical and surgical tables merely show the numbers admitted, with the various diseases tabulated, and give no results. The post-mortem table is carefully compiled, and gives all necessary information of the deaths. The same may be said of the operation table, which shows the numbers cured, relieved, unrelieved, and the deaths. For instance, we notice that the operation for appendicitis was performed 14 times with 1 death; ovariotomy 5 times with 1 death; laparotomy 3 times for ectopic pregnancy, all being successful. No anæsthetic table is given. In the ophthalmic department 1,744 patients were treated, and of 128 operations herein performed only 1 was unsuccessful. Chloroform was administered 41 times. In the open-air wards for consumption 19 cases were treated, with the result that in 4 instances there was complete arrest of the disease, in 7 there was much improvement, in 5 some improvement, in 1 there was no improvement, 1 became worse, and 1 died. When such results can be obtained in wards not specially built for the purpose, and perhaps not well adapted for it, some idea may be formed of the advantages of modern treatment for this scourge, and we echo the words of the report that this expedient "should only be a temporary measure, preliminary to the construction of a sanatorium for the town and county." With new pavilions for the ordinary sick, with departments for eye diseases, and for consumption, and with properly equipped laboratories, there is no reason why the institution should not become, to some extent at least, a medical school. From this two advantages are always forthcoming—increased income to the institution and increased efficiency in the medical and surgical staff. To have to teach others is the best preservative of the knowledge already possessed and the best stimulus to increase that knowledge.

